# Radiologic Predictors for Clinical Stage IA Lung Adenocarcinoma with Ground Glass Components: A Multi-Center Study of Long-Term Outcomes

**DOI:** 10.1371/journal.pone.0136616

**Published:** 2015-09-04

**Authors:** Zhao Li, Bo Ye, Minwei Bao, Binbin Xu, Qinyi Chen, Sida Liu, Yudong Han, Mingzhen Peng, Zhifeng Lin, Jingpei Li, Wenzhuo Zhu, Qiang Lin, Liwen Xiong

**Affiliations:** 1 Department of Thoracic Surgery, Shanghai General Hospital, Shanghai Jiaotong University School of medicine, Shanghai, China; 2 Department of Thoracic Surgery, Shanghai Chest Hospital, Shanghai Jiaotong University School of Medicine, Shanghai, China; 3 Department of Thoracic Surgery, Shanghai Pulmonary Hospital, Shanghai Tongji University School of Medicine, Shanghai, China; 4 Department of Dermatology, Huashan Hospital, Fudan University, Shanghai, China; 5 Department of Thoracic Surgery, Guangzhou Medical University First Affiliated Hospital, Guangdong Province, China; University of North Carolina School of Medicine, UNITED STATES

## Abstract

**Objective:**

This study was to define preoperative predictors from radiologic findings for the pathologic risk groups based on long-term surgical outcomes, in the aim to help guide individualized patient management.

**Methods:**

We retrospectively reviewed 321 consecutive patients with clinical stage IA lung adenocarcinoma with ground glass component on computed tomography (CT) scanning. Pathologic diagnosis for resection specimens was based on the 2011 IASLC/ATS/ERS classification of lung adenocarcinoma. Patients were classified into different pathologic risk grading groups based on their lymph node status, local regional recurrence and overall survival. Radiologic characteristics of the pulmonary nodules were re-evaluated by reconstructed three-dimension CT (3D-CT). Univariate and multivariate analysis identifies independent radiologic predictors from tumor diameter, total volume (TV), average CT value (AVG), and solid-to-tumor (S/T) ratio. Receiver operating characteristic curves (ROC) studies were carried out to determine the cutoff value(s) for the predictor(s). Univariate cox regression model was used to determine the clinical significance of the above findings.

**Results:**

A total of 321 patients with clinical stage IA lung adenocarcinoma with ground glass components were included in our study. Patients were classified into two pathologic low- and high- risk groups based on their distinguished surgical outcomes. A total of 134 patients fell into the low-risk group. Univariate and multivariate analyses identified AVG (HR: 32.210, 95% CI: 3.020–79.689, P<0.001) and S/T ratio (HR: 12.212, 95% CI: 5.441–27.408, P<0.001) as independent predictors for pathologic risk grading. ROC curves studies suggested the optimal cut-off values for AVG and S/T ratio were-198 (area under the curve [AUC] 0.921), 2.9 (AUC 0.996) and 54% (AUC 0.907), respectively. The tumor diameter and TV were excluded for the low AUCs (0.778 and 0.767). Both the cutoff values of AVG and S/T ratio were correlated with pathologic risk classification (p<0.001). Univariate Cox regression model identified clinical risk classification (RR: 3.011, 95%CI: 0.796–7.882, P = 0.095) as a good predictor for recurrence-free survival (RFS) in patients with clinical stage IA lung adenocarcinoma. Statistical significance of 5-year OS and RFS was noted among clinical low-, moderate- and high-risk groups (log-rank, p = 0.024 and 0.010).

**Conclusions:**

The AVG and the S/T ratio by reconstructed 3D-CT are important preoperative radiologic predictors for pathologic risk grading. The two cutoff values of AVG and S/T ratio are recommended in decision-making for patients with clinical stage IA lung adenocarcinoma with ground glass components.

## Introduction

The introduction of the high-resolution CT (HRCT) scanning has greatly enhanced early detection of small-sized lung adenocarcinoma with remarkable reduction of mortality from lung cancers [1]. Pulmonary nodules with a wide area of ground-glass components are normally believed to have an excellent prognosis due to their minimally invasive nature [2, 3]. However, nodules with less than 50% ground glass area are at greater risk of lymph node metastasis and poor prognosis. The international multidisciplinary classification of lung adenocarcinoma indicated great discrepancy in prognosis for different pathological subtypes of clinical stage IA lung cancers [4–8]. For example, the 5-year disease-free survival (DFS) for adenocarcinoma in situ and minimally invasive adenocarcinoma was 100%. However, for micropapillary- and solid-predominant adenocarcinoma, the 5-year DFS was between 67 and 76%, respectively [9]. Due to the heterogeneity of the tumors and their different responses to surgical management, there is a great need to identify which patients could benefit most from major resection and lymph node resection.

However, decisions of pathologic diagnosis based on intra-operative frozen section could be difficult to exclude tumor malignancy and lymph node metastasis [9]. The guideline of American Thoracic Society (ATS) recommended that the solid component and the total tumor size on radiologic findings could help in preoperative differentiation of tumor malignancy [10]. Recent studies found that the ratio of solid areas to ground glass components could be used to determine the invasiveness of clinical stage IA lung adenocarcinomas [2, 11, 12]. Similarly, the CT value has been reported as another indicator for evaluating aggressiveness of nodules with ground glass components [11, 13, 14]. However, these radiologic data were partially subjective due to the measurement of only one tumor slice at the largest cut surface.

Multi-detector CT scanning is now widely used in routine practice and three-dimensional (3D) constructive CT enables volumetric data for pulmonary nodules. Volumetric measurements on a three-dimensional scale have been implicated as more accurate compared to conventional measurements on a one- or two-dimensional scale [15, 16]. The purpose of this study was to define the pathohistologic risk grading based on long-term surgical outcomes. We then correlated this pathologic classification with preoperative radiologic findings from 3D-CT. Statistical analysis was performed to figure out the optimal cut-off values of preoperative radiologic predictors for defining the pathological risk grading of clinical IA adenocarcinoma with ground glass components in the aim to guide individualized patient management.

## Patients and Methods

Between 2006 and 2010, 14,022 consecutive patients with non-small cell lung cancer (NSCLC) underwent surgical resection at Shanghai Chest Hospital Shanghai, Shanghais General Hospital and Shanghai 411 Hospital.

Patients were included in this study if they had (1) clinical stage IA lung adenocarcinoma with ground glass components on thin-section computed tomography (CT) scanning, (2) pulmonary lobectomy and systematic lymph node dissection. We identified 726 patients with clinical stage IA lung adenocarcinoma who met these criteria. Patients were excluded if they had (1) lymph node positivity on CT scanning or distant metastasis (n = 151), (2) neoadjuvant chemotherapy or radiotherapy (n = 120), and (3) solid or pure ground glass opacity (GGO) nodules based on Suzuki classification (n = 134) [17]. The remaining 321 patients were included in the study.

This retrospective study was begun in November, 2014, after receiving approval from the Clinical Ethics Committee. Prior written informed consent was obtained from all the patients. Medical record data and tissue specimens were anonymized prior to analysis. Patient identities were known only to Zhao Li. Study protocol was approved by the local Institutional Review Board and the Clinical Ethics Committee of Shanghai General Hospital, Shanghai Jiaotong University school of medicine, whose approval covered the research program at Chest Hospital and 411 Hospital.

## Pathologic Evaluation

Resection specimens were examined by two independent pathologists. Both were blinded to CT findings. Tumor subtypes were classified according to the 2011 international multidisciplinary classification of lung adenocarcinoma [18]. Pathologic node stage was categorized as pN0, pN1, or pN2.

## Radiologic Analysis and Follow-Ups

**Fig 1:**
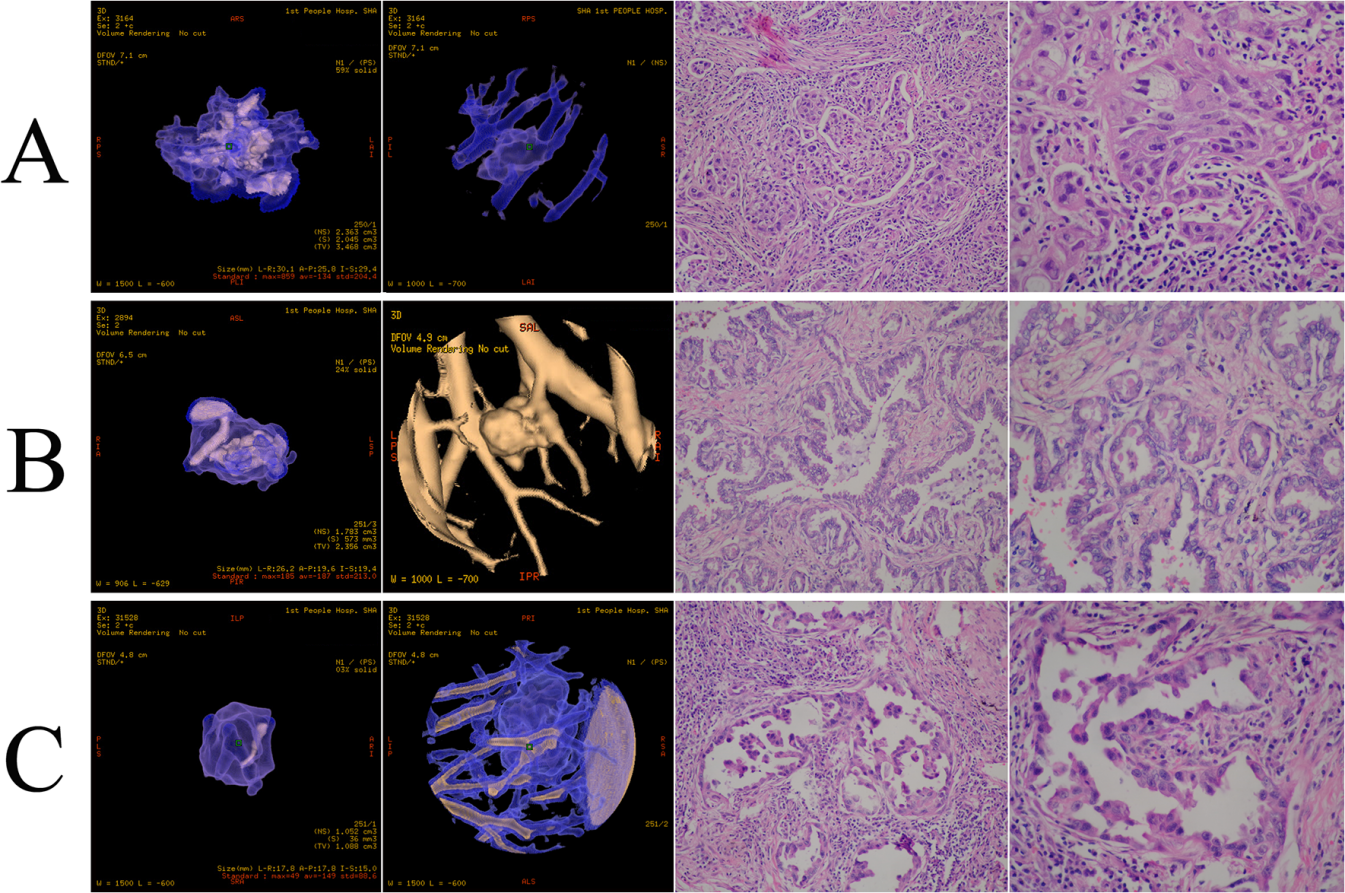
Three-dimensional computed tomography (3D-CT) of clinical stage IA lung adenocarcinoma with ground glass component. (A) AVG = -134, S/T ratio = 59%, postoperative pathologic subtype: solid predominant adenocarcinoma (SPA); (B) AVG = -187, S/T ratio = 24%, postoperative pathologic subtype: acinar predominant adenocarcinoma (APA); (C) AVG = -149, S/T ratio = 63%, postoperative pathologic subtype: micropapillary predominant adenocarcinoma (MPA). All parameters of the 3D-CT were computed automatically after the operator placed a marker on the nodule. All postoperative specimens were reexamined by two pathologists.

The three-dimensional (3D) high-resolution images of the lung lesions were reconstructed by two independent radiologists using a computerized automated diagnosis (CAD) system, which has been reported previously[19, 20]. Both were blinded to pathologic findings. The tumor diameter was defined as the maximum tumor diameter and determined digitally by the CT scan lung window. The solid-to-tumor (S/T) ratio was defined as the proportion of the maximum solidation (S) volume divided by the maximum tumor (T) volume. The tumor diameter, total volume (TV), average CT value (AVG) were measured on a commercially available workstation (Advantage Workstation 4.3; GE Healthcare) with CT lung analysis software (Lung VCAR; GE Healthcare). The S/T ratio was computed automatically after the operator placing marker on the nodule (Fig 1) [20–23].

CT scans were performed post-operatively at month 3, 6 and annually thereafter to determine local regional recurrence. Disease-free survival (DFS) was defined as the period from the date of operation until any recurrence, death from any cause, or the end of the follow-up.

## Surgical Procedures

All patients underwent pulmonary lobectomy and systematic lymph node dissection. A wedge resection of the primary tumor was sent for frozen section before performing nodal dissection. Patients who underwent only wedge resection due to poor lung function were not included in this study. Systematic lymph node dissection was performed in the same manner in all patients, including the removal of all lymphatic tissues within the defined anatomic landmarks of stations 2, 4, and 7 to 12 on the right and stations 4 to 12 on the left, according to the classification of the American Thoracic Society. Lymph node dissection complied with the recommendations of the European Society of Thoracic Surgeons[24]. At least 6 nodes were removed in all patients, including intrapulmonary, hilar, and mediastinal nodes.

## Statistical Analysis

Student’s t tests or the Wilcoxon rank-sum test, depending on the normality of distribution, and the x^2^ test or Fisher’s exact test were used to compare continuous and categorical variables, respectively.

Receiver-operating characteristic (ROC) curves were used to determine the optimal cutoff points of the radiologic predictors for pathologic risk grading. Univariate and multiple logistic regression analysis were performed to identify the independent radiologic predictors for pathologic risk.

The overall survival (OS) and DFS curves were calculated using the Kaplan-Meier method, and differences in the curves were determined using the log-rank test. Multivariate analysis of DFS was performed with Cox’s proportional hazard model. Statistical analyses were performed using SPSS Statistics, version 17.0 (SPSS Inc, Chicago, IL).

## Results

### Clinical and pathologic characteristics

Table 1 summarizes the clinical and pathologic characteristics of the 160 (49.8%) female and 161 (50.2%) male patients (mean age of 53.93 years, ranging from 28 to 76 years) with clinical stage IA lung adenocarcinoma. A median of 10.3 of lymph node stations were sampled in all 321 patients. Lymph node metastasis was present in 29 patients (12.6%), including 16 (6.9%) with pN1 disease and 13 (5.6%) with pN2 disease. The mean follow-up period was 58.94 months (ranging from 22 to 93 months).

**Table 1:** Relation of clinical and pathologic factors with lymph node metastasis and postoperative recurrence in 321 patients with clinical stage IA lung adenocarcinoma.

	Lymph node status				Postoperative recurrence		
Variable	*P*N_0_	*P*N_1_	*P*N_2_	*P* ^*a*^	Yes	No	*P* ^*a*^
Age (≥ 48 / <48 yrs)	116/145	6/29	2/23	<0.001	5/16	119/181	0.149
Sex (Male/Female)	130/131	19/16	12/13	0.862	8/13	153/147	0.269
Smoking (never/current or former)	135/126	15/20	17/8	0.154	11/10	156/144	0.557
Pathologic subtype	261	35	25	<0.001	21	300	<0.001
Atypical adenomatous hyperplasia	17	0	0		0	17	
Adenocarcinoma in situ	17	0	0		0	17	
Minimally invasive adenocarcinoma	29	0	0		0	29	
Lepidic predominant adenocarcinoma	19	2	0		3	18	
Papillary predominant adenocarcinoma	43	0	0		0	42	
Invasive mucinous adenocarcinoma	7	1	0		1	7	
Micropapillary predominant adenocarcinoma	20	6	2		1	27	
Solid predominant adenocarcinoma	25	7	9		12	29	
Acinar predominant adenocarcinoma	85	19	14		4	114	
Pathologic low-risk group1	131	3	0	<0.001	4	130	0.029
Pathologic high-risk group2	130	32	25		17	170	

^a^Fisher exact test or X^2^ test.

Pathologic low-risk group consisted of atypical adenomatous hyperplasia, adenocarcinoma in situ, minimally invasive, lepidic predominant, papillary predominant and invasive mucinous. Pathologic high-risk group included micropapillary predominant, solid predominant and acinar predominant.

Table 1 compared each pathologic subtype based on lymph node status and postoperative recurrence. The Fisher exact test showed that the histopathologic subtype was significantly associated with lymph node metastasis and postoperative recurrence (P<0.001).

### Pathologic risk classification

**Fig 2:**
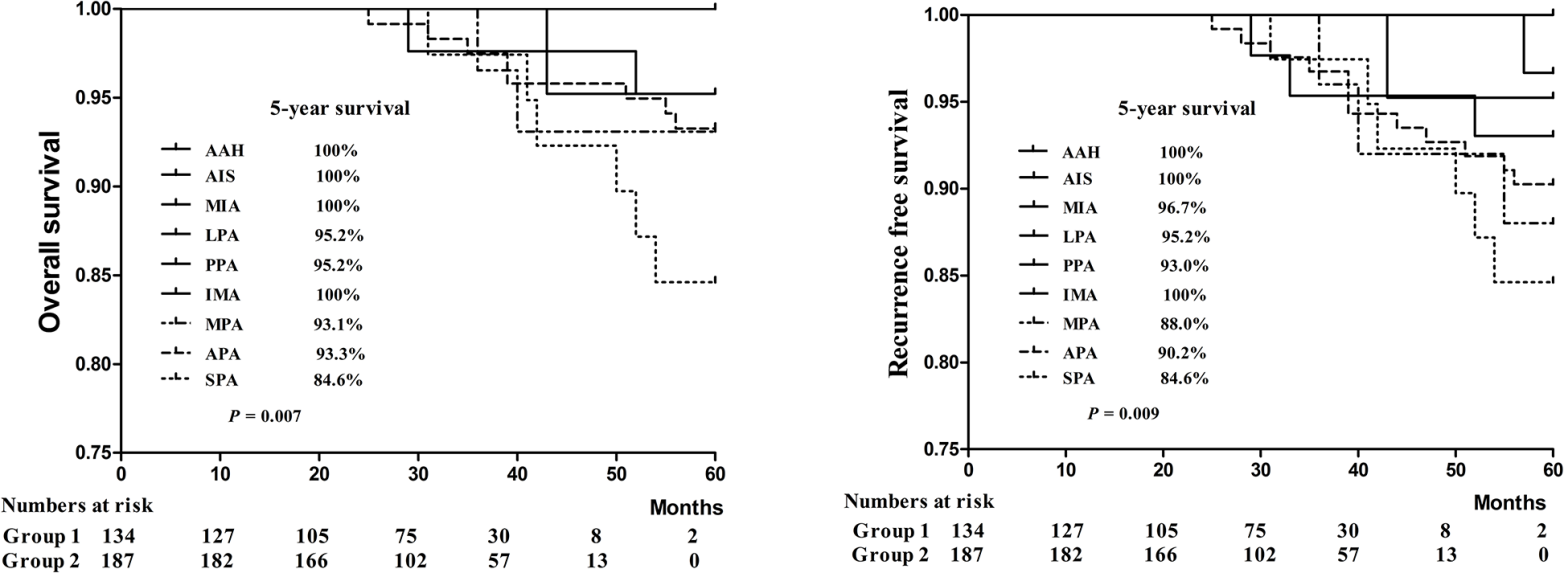
Overall survival (OS) and recurrence-free survival (RFS) curves for patients with different postoperative pathologic subtypes. Group 1, or low-risk group, contained the atypical adenomatous hyperplasia, adenocarcinoma in situ, minimally invasive, lepidic predominant, papillary predominant and invasive mucinous subgroups. Group 2, or high-risk group, contained the micropapillary predominant, solid predominant and acinar predominant subgroups.

The OS and RFS of the patients with different pathologic subtypes were shown in Fig 2. The 5-year OS rate of the patients with solid predominant lung adenocarcinoma was significantly low (OS = 84.6%, P = 0.007). The 5-year RFS of the patients with micropapillary predominant (RFS = 88.0%), solid predominant (RFS = 84.6%) and acinar predominant lung adenocarcinoma (RFS = 90.2%) were lower than the patients with other pathologic subtypes (P = 0.009). We accordingly classified the patients into two groups, i.e. group 1, or pathologic low-risk group (atypical adenomatous hyperplasia, adenocarcinoma in situ, minimally invasive, lepidic predominant, papillary predominant and invasive mucinous) and group 2, or high-risk group (micropapillary predominant, solid predominant and acinar predominant).

Lymph node involvement was noted in 57 patients (17.8%) in the pathologic high-risk group (32 with pN1 and 25 with pN2) and 3 patients (0.9%) with pN1 in the pathologic low-risk group. Four patients (1.2%) in the pathologic low-risk group and 17 patients (5.3%) in the pathologic high-risk group had postoperative recurrence (Table 1).

### Univariate and multivariate analyses for independent predictors of pathologic risk grading

Univariate analyses showed the tumor diameter, TV, AVG and S/T ratio as significant determinants. However, multivariate analysis identified that AVG (HR: 32.210, 95% CI: 3.020–79.689, P<0.001) and S/T ratio (HR: 12.212, 95% CI: 5.441–27.408, P<0.001) were significantly independent predictors for pathologic risk grading (Table 2).

**Table 2:** Independent predictors for pathologic risk grading.

	Univariate			Multivariate		
Variable	Low-risk^a^	High-risk^b^	*P*	HR	95% CI	*P*
Tumor diameter (≥1.7 / <1.7)	48/86	128/59	*P*<0.001	1.031	0.444-2.397	0.943
AVG (≥-198 / <-198)	26/108	178/9	*P*<0.001	32.210	3.020-79.689	*P*<0.001
S/T ratio (≥54% / <54%)	17/117	149/38	*P*<0.001	12.212	5.441-27.408	*P*<0.001
TV (≥1.77 / <1.77)	55/79	155/32	*P*<0.001	2.690	1.026-7.055	0.044

CI = confidence interval; HR = hazard ratio.

^a^ pathologic low-risk group

^b^ pathologic high-risk group.

### Receiver-operating characteristic (ROC) curves for predicting pathologic risk grading

**Fig 3:**
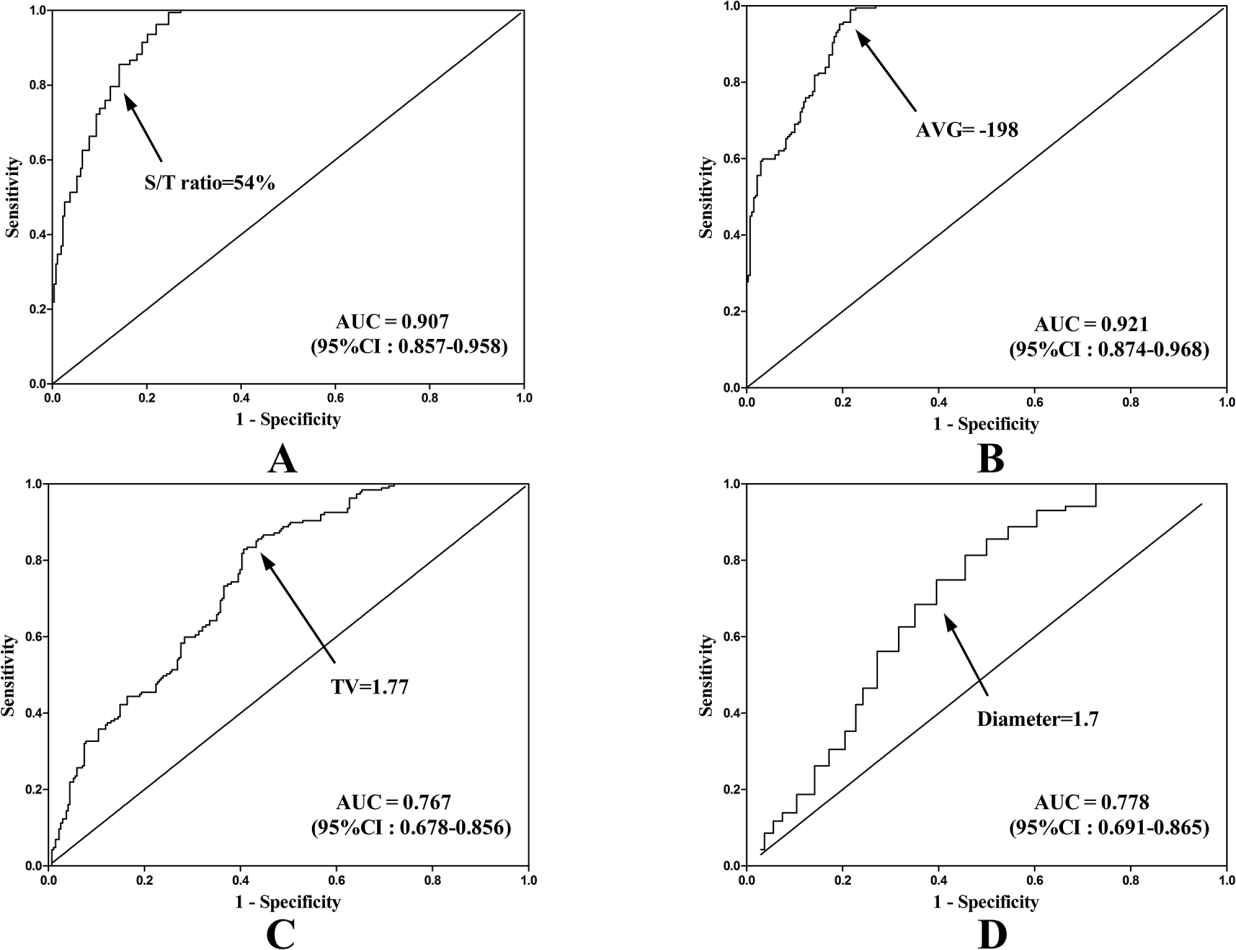
Area under the curve (AUC) of the receiver-operating characteristics (ROC) for differentiation of pathologic high- and low-risk group. (A) solid-to-tumor (S/T) ratio: AUC, 0.921 (95%CI: 0.857–0.958). (B) average CT value (AVG): AUC, 0.921 (95%CI: 0.857–0.958). (C) Total volume (TV): AUC, 0.767 (95% CI: 0.678–0.856). (D) Tumor diameter: AUC, 0.778 (95% CI:0.691–0.865).

The ROC curves for predicting the pathologic high-risk group and calculating the optimal cut-off values of AVG and S/T ratio were-198 (area under the curve [AUC] 0.921) and 54% (AUC 0.907), respectively (Fig 3). Tumor diameter and TV were excluded due to the low AUC (0.778 and 0.767). Both of the cutoff values of AVG and S/T ratio were statistically correlated with pathologic risk grading (p<0.001).

### Clinical risk classification

We then classified the patients based on the S/T ratios and AVGs into the three following groups, namely, clinical low-risk group (S/T ratio < 54% and AVG < -198, n = 102), clinical moderate-risk group (S/T ratio ≥ 54% and AVG < -198, or S/T ratio < 54% and AVG ≥ -198, n = 68), and clinical high-risk group (S/T ratio ≥ 54% and AVG ≥ -198, n = 151). A Univariate Cox regression model identified this clinical risk classification (RR: 3.011, 95%CI: 0.796–7.882, P = 0.095) as a good predictor for DFS in patients with clinical stage IA lung adenocarcinoma (Table 3).

**Table 3:** Comparisons of the clinical classification with lymph node metastasis and a Univariate Cox regression model of clinical risk classification.

	Lymph node metastasis		Cox Regression	
Variables	N0 N1 N2	*P*	RR (95% CI)	*P*
Clinical classification		P<0.001	3.011 (0.796-7.882)	0.095
Low-risk	101 / 1 / 0			
Moderate-risk	55 / 10 / 3			
High-risk	105 / 24 / 22			

**Fig 4:**
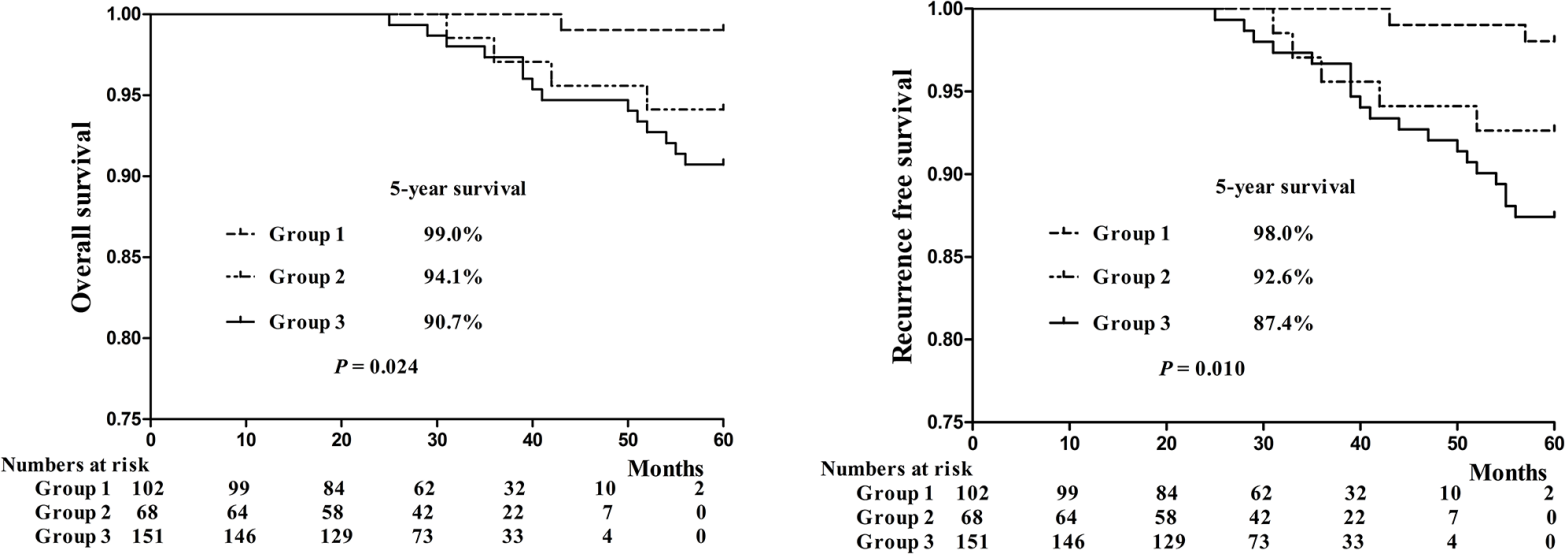
Overall survival (OS) and recurrence-free survival (RFS) curves for the patients with clinical stage IA lung adenocarcinoma with ground-glass components who underwent surgical resection in group1, group2 and group 3. Group 1 = clinical high-risk group, group 2 = clinical moderate-risk group, group 3 = clinical low-risk group.

The OS and RFS of the patients in these three groups were shown in Fig 4. The 5-year OS rates for patients of clinical high-, moderate- and low-risk groups were 90.7%, 94.1% and 99.0%, respectively. The 5-year RFS of the patients in the clinical high-, moderate- and low-risk groups were 87.4%, 92.6% and 99.0%, respectively. Statistical significance was noted among the three groups in terms of long-term surgical outcomes (log-rank, p = 0.024 and 0.010).

## Discussion

The current surgical management for clinical stage IA lung adenocarcinoma remains lobectomy and systematic lymph node dissection for intra-operative malignant pathologic findings [25–27]. However, pathological and radiological studies revealed significant prognostic subsets despite standardized surgical approaches [18, 28–31]. The new classification of lung adenocarcinoma proposed by IASLC/ATS/ERS in 2011 supported the idea of identification of histologic subsets to guide treatment decision-making [32]. Here we retrospectively reviewed 321 patients with clinical stage IA lung adenocarcinoma. Our data confirmed significant differences in surgical outcomes based on the new guideline. In our study, pathologic subtypes fell into two major prognostic categories based on lymph node metastasis, recurrence and overall survival. The pathologic low-risk group consisted of atypical adenomatous hyperplasia, adenocarcinoma in situ, minimally invasive, lepidic predominant, papillary predominant and invasive mucinous. Such patients could be candidates for limited surgical resection in the future. Mediastinal lymph node dissection or sampling was reported to be associated with 38% total morbidity [4, 26]. It is possible that such morbidity might be prevented by limited surgical resection. On the other hand, patients with micropapillary predominant, solid predominant and acinar predominant subtypes were of significantly greater risk of lymph node metastasis, local region recurrence and poorer overall survival. There is growing data showing that the solid subtype correlates with poor outcomes [9, 18, 32]. Therefore, there is a great need to identify which patients are of probable poor prognosis in order to determine which patients may benefit most by standardized surgical treatment. However, it can be very difficult for pathologists to exclude invasiveness on the basis of intra-operative frozen section [9]. To our knowledge, this study was for the first time to correlate pre-operative radiological findings with the new pathologic histologic classification, where clear cutoff points of radiological parameters were provided for individualized patient management.

The introduction of computed tomography (CT) scan has greatly enhanced the detection of early lung cancer [3, 33]. More recent studies have been focused on the differentiation of small-sized nodules (less than 20 mm in diameter), especially nodules with ground glass components, to predict lymph node metastasis and recurrence [8, 34, 35]. Given the heterogeneity nature of clinical stage IA adenocarcinoma, Suzuki et al classified the small-sized lung adenocarcinoma into six subtypes by radiologic findings based on tumor diameter, solid or ground glass components and heterogeneity of the tumor [36]. In previous studies, the most frequently used radiologic indicators included tumor diameter and areas of nodules [37–39]. However, measurement of these values is semiquantitative and limited given the irregular shape of the solid portions of the nodules. Volumetric measurement of pulmonary nodules on a three-dimension scale by digital scanners has been reported to enable more objective and accurate quantification of pulmonary ground glass nodules [22]. Further investigations showed that the ratio of solid component to whole nodule volume or S/T ratio was critical in evaluating the progression of ground glass nodules [13].

In our study, S/T ratio and AVG, not tumor diameter or tumor volume, turned out to be independent predictors for pathologic risk subsets. These values were achieved on a three-dimensional scale and reconstructed automatically by software (Lung VCAR, GE Healthcare) using volume rendering on the specific workstation (Advantage Workstation 4.3, GE Healthcare) [20–23]. The measurement is thus reproducible and more objective, which is critical for further analysis of the nodules. The area of ground glass component as less than 50% has been believed as a cutoff point in identifying a candidate for poor prognosis and major resection. However, this value was based on a two-dimensional semiquantitation to simplify visual judgment. To our knowledge, there has been no data available yet for a more reliable and quantified assessment of radiologic findings correlated to the new pathologic classification. In our study, the receiving operating characteristic (ROC) curve analysis found optimal cutoff values of AVG and S/T ratio were-198 and 54%, respectively, for pathologic risk-grading prediction. Area under the curve (AUC) for AVG and S/T ratio were 0.921 and 0.907, respectively, statistically indicating high diagnostic value for these two values. The clinical significance of the two cutoff values was further explored by classifying patients into three clinical risks grading accordingly. Overall survival (OS) and disease free survival (DFS) in high- and moderate-risk group were significantly lower compared to those in the low-risk group. Based on these findings, we believe reconstructed 3D-CT radiography is necessary for patients having clinical stage IA lung adenocarcinoma with ground glass components. Patients who fall into the clinical high- and low-risk groups were strongly recommended for standardized surgical resection and systemic lymph node dissection. Meanwhile, for patients in the low-risk group limited resection, especially wedge resection, and long-interval follow-ups are recommended.

This retrospective study was limited in certain aspects. First, the operations were performed by different surgeons with varying surgical approaches, including video-assisted thoracic surgical procedures and open surgical procedures. Additionally, the heterogeneity of the pathologic diagnosis was inevitable to some extent. The parameter measurement of the 3D-CT findings had significant differences on inspiratory and expiratory scans. Further prospective studies should be performed to determine the validality of the preoperative radiologic prediction of histopathologic risk grading based on present clinical risk classification.

## Conclusions

The AVG and the S/T ratio by reconstructed 3D-CT are important preoperative radiologic predictors for pathologic risk grading. The two cutoff values of AVG and S/T ratio are recommended in decsion-making for patients with clinical stage IA lung adenocarcinoma with ground glass components.

## Supporting Information

S1 File PLOS ONE Clinical Studies Checklist. (PDF)

S2 File STROBE Statement. All items of the checklists were included in the reports of our observational studies.
(PDF)

S3 File A translated version of research ethics approval. The research ethics approval from Shanghai First People's Hospital ethics committee was translated into English.
(PDF)

S4 File Original copy of research ethics approval. The research ethics approval came from Shanghai First People's Hospital ethics committee.
(PDF)
