# Supplementary material for: Radiologic Predictors for Clinical Stage IA Lung Adenocarcinoma with Ground Glass Components: A Multi-Center Study of Long-Term Outcomes
Source: PLoS One. 2015 Sep 4;10(9):e0136616. doi: 10.1371/journal.pone.0136616 (PMC4560441; doi:10.1371/journal.pone.0136616)
Supplement: S1 File — (PDF) [file pone.0136616.s001.pdf]

PLOS ONE Clinical Studies Checklist

1. For at least one author, provide an institutional email address for correspondence.

lizhao19881228@163.com

2. Describe any previous interactions you have had with *PLOS ONE* in relation to this work.

/

3. If submitting a systematic review or meta-analysis, please describe recent related systematic reviews and meta-analyses, and how the current study adds to the field in the context of previous reports.

/

4. Please explain the rationale for your study.

This multi-centre study analyzed the relationship between the radiologic findings of GGO nodules from 3D-CT and new pathologic types in the assessment of survival and postoperative recurrence in clinical stage IA lung adenocarcinoma. We aimed to address guidelines for the preoperative pathologic diagnosis and the selection of the operation time and procedure for early stage lung cancer.

HUMAN SUBJECTS RESEARCH

Please complete #5-9 if your study involved any human participants or human subjects' data, including medical record data. These questions should be addressed for prospective and retrospective studies.

5. If you did not have ethics approval, please explain why this was not necessary.

   We had.

6. Upload the letter of ethical approval from your ethics committee as file type "Other". If the letter is not in English, please include a translation.

   __Y_ Uploaded ___ N/A

7. Upload a copy of the study protocol approved by your ethics committee as file type "Other" – if the protocol is not in English, please include a translation.

___ Uploaded __N_ N/A (if N/A, provide explanation below)

My research program was described detailedly in the Patients and Methods section. We received the ethical approval and did not got the study protocol approval.

8. If the study involved patients and/or patient medical data, report the date range within which patients were recruited to the study in the Methods section.

__Y_ Completed ___ N/A

9. If the study included patients/participants, include details on sample size calculation and power analysis below and in the Methods section.

_Y__ Completed ___ N/A

REPORTING GUIDELINES

For the relevant study type, upload the applicable reporting checklist and other documents listed as Supporting Information files for your submission. Note: in most cases, only one study type will be relevant.*

10. ***Clinical Trial***
  - CONSORT flow diagram
  - Completed CONSORT or TREND checklist
  - Include details on clinical trial registration in your Methods section.
  - Completed TIDieR checklist

___ Uploaded ___ N/A

11. ***Observational study***
  - Completed STROBE checklist

__Y_ Uploaded ___ N/A

12. • ***Meta-analysis or Systematic Review*** PRISMA flowchart (this should be Figure 1 of your manuscript)
 • Completed PRISMA checklist

___ Uploaded ___ N/A

****URLs for reporting checklists and guidelines:***

CONSORT (randomized trials): http://www.consort-statement.org/consort-statement/checklist

CONSORT flow diagram: http://www.consort-statement.org/consort-statement/flow-diagram

TREND (non-randomized trials): http://www.cdc.gov/trendstatement/

STROBE: http://www.strobe-statement.org/index.php?id=available-checklists

PRISMA: http://www.prisma-statement.org/statement.htm

TIDieR: http://www.equator-network.org/reporting-guidelines/tidier/
