# Supplementary material for: Radiologic Predictors for Clinical Stage IA Lung Adenocarcinoma with Ground Glass Components: A Multi-Center Study of Long-Term Outcomes
Source: PLoS One. 2015 Sep 4;10(9):e0136616. doi: 10.1371/journal.pone.0136616 (PMC4560441; doi:10.1371/journal.pone.0136616)
Supplement: S3 File — The research ethics approval from Shanghai First People's Hospital ethics committee was translated into English. (PDF) [file pone.0136616.s003.pdf]

# Research ethics approval of Shanghai First People's Hospital ethics committee

| Approval Document No. 2014KY117 | | Review date 2014.11.11 | Item Number 2014.114 |
|---|---|---|---|
| Project Title | Clinical research of predictive factors for pathologic features in patients with clinical stage IA Non–Small Cell Lung Cancer | | |
| Project Source | Shanghai First People's Hospital, Affiliated Shanghai Jiaotong University | | |
| Principal Investigator | Qiang Lin | | |
| Research Unit | Shanghai First People's Hospital, Affiliated Shanghai Jiaotong University | Department | thoracic surgery |

Review documents (including version number) as follows:

(1) research program

(2) Informed Consent

(3) the principal investigator resume

1. Review mode

☐ meeting review ■ quick review ☐ emergency meeting review

2, Result of the review

Agree

3, whether the project would be reviewed of the research ethics committee continuously?

☐ Yes ■ No

4, this document is valid for one year, expiring on December 31, 2015.

Medical Ethics Committee (stamp)

Date: November 12, 2014

Address: Haining Road 100(200080) Tel: (021) 63240090
