# Supplementary material for: Radiologic Predictors for Clinical Stage IA Lung Adenocarcinoma with Ground Glass Components: A Multi-Center Study of Long-Term Outcomes
Source: PLoS One. 2015 Sep 4;10(9):e0136616. doi: 10.1371/journal.pone.0136616 (PMC4560441; doi:10.1371/journal.pone.0136616)
Supplement: S4 File — The research ethics approval came from Shanghai First People's Hospital ethics committee. (PDF) [file pone.0136616.s004.pdf]

上海市第一人民医院伦理委员会科研项目伦理批件

批件编号 2014KY117		审查日期 2014.11.11	项目编号 2014科114
项目名称	临床IA期非小细胞肺癌的病理特征的预测因素的临床研究		
项目来源	上海交通大学附属第一人民医院		
主要研究者	林强		
研究单位	上海交通大学附属第一人民医院	科室	胸外科

审查文件（含版本号）如下：

（1） 研究方案

（2） 知情同意书

（3） 主要研究者履历

1、审查方式

☐会议审查　■快速审查　☐紧急会议审查

2、审查结果

同意

3、该研究进行过程中将受伦理委员会的持续审查？☐是　■否

审查频率为该研究批准之日起每____月一次

4、批件有效期为__一__年，至__2015年12月31号__止。

医院伦理委员会（盖章）

日期：2014年11月12日

地址：海宁路100号 （200080）　电话：（021）63240090
